# Mapping Informative Clusters in a Hierarchial Framework of fMRI Multivariate Analysis

**DOI:** 10.1371/journal.pone.0015065

**Published:** 2010-11-30

**Authors:** Rui Xu, Zonglei Zhen, Jia Liu

**Affiliations:** 1 College of Life Science, Graduate University of Chinese Academy of Sciences, Beijing, People's Republic of China; 2 State Key Laboratory of Cognitive Neuroscience and Learning, Beijing Normal University, Beijing, People's Republic of China; Indiana University, United States of America

## Abstract

Pattern recognition methods have become increasingly popular in fMRI data analysis, which are powerful in discriminating between multi-voxel patterns of brain activities associated with different mental states. However, when they are used in functional brain mapping, the location of discriminative voxels varies significantly, raising difficulties in interpreting the locus of the effect. Here we proposed a hierarchical framework of multivariate approach that maps informative clusters rather than voxels to achieve reliable functional brain mapping without compromising the discriminative power. In particular, we first searched for local homogeneous clusters that consisted of voxels with similar response profiles. Then, a multi-voxel classifier was built for each cluster to extract discriminative information from the multi-voxel patterns. Finally, through multivariate ranking, outputs from the classifiers were served as a multi-cluster pattern to identify informative clusters by examining interactions among clusters. Results from both simulated and real fMRI data demonstrated that this hierarchical approach showed better performance in the robustness of functional brain mapping than traditional voxel-based multivariate methods. In addition, the mapped clusters were highly overlapped for two perceptually equivalent object categories, further confirming the validity of our approach. In short, the hierarchical framework of multivariate approach is suitable for both pattern classification and brain mapping in fMRI studies.

## Introduction

Multi-voxel pattern analysis (MVPA) methods have been widely used in fMRI studies to characterize the relationship between fMRI responses and cognitive functions. In a typical MVPA framework, a multivariate classifier is trained on multi-voxel patterns of brain activities with known labels, and the trained classifier is then used to classify untrained data (for a review, see [1], [2], [3]). The MVPA methods have been proved successful in differentiating stimulus categories or task states in both human [4], [5], [6], [7], [8], [9] and monkey fMRI studies [10]. Recently, interests have been dedicated to reliably localizing such discriminative information in the brain [3]. Here we proposed a new approach based on clusters rather than voxels for this purpose.

One of the core objectives in functional neuroimaging is to link cortical regions to cognitive functions. Accordingly, a univariate statistical parametric mapping approach has been proposed [11], [12]. However, the univariate approach is unable to extract information embedded in multi-voxel patterns because each voxel is treated independently. To address this issue, multivariate feature selection algorithms from machine learning have been used in analyzing multi-voxel patterns of brain activation [13], [14], [15], [16], [17], [18]. These algorithms mainly aim to select a minimum set of voxels necessary for constructing a classifier with the best predictive accuracy [19]. However, because extensive spatiotemporal correlations in fMRI responses among neighboring voxels lead to high redundancy of features, only a small set of voxels with similar response profiles are selected. This leads to two problems that limit the application of voxel-based MVPA methods in functional brain mapping. First, the selected voxels are usually distributed rather than clustered, some of which may be present outside the brain (e.g. [15], but see [16], [18]). Second, small variations of data may cause completely different sets of voxels being selected [17].

To tackle these problems, we used local homogeneous clusters [20], [21], not individual voxels, as basic units for the brain mapping in the MVPA. To this end, we proposed a new approach where both brain activation patterns within local homogeneous clusters and the interaction among these clusters were examined. We termed this approach as mapping informative clusters (MIC), in contrast to traditional voxel-based approaches of mapping informative voxels (MIV). Results from both simulated and real fMRI data showed that our MIC method outperformed the MIV method in localizing informative regions while the predictive accuracy was largely preserved.

## Methods

**Figure 1:**
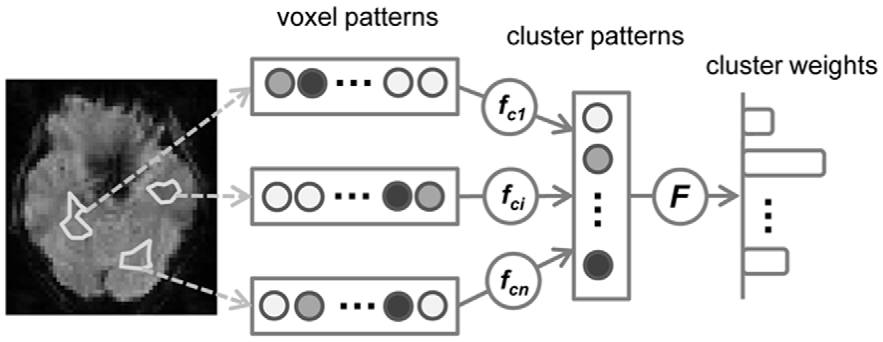
The framework of the mapping informative clusters (MIC) approach. The whole brain was partitioned into homogeneous clusters, where the within-cluster voxel patterns from different clusters were transformed into a cluster pattern consisted of one single value for each cluster. Informative clusters were selected according to their discriminative weights derived from the cluster pattern.

In the MIC, a hierarchical framework was used to identify the most informative clusters by examining both local and global patterns of fMRI data (Figure 1). First, through a multi-voxel classifier, the multi-voxel pattern within a homogeneous cluster was summarized to a single value that represented condition-related information carried by the cluster. The output values of all clusters were then taken as a multi-cluster pattern to construct a second-level multi-cluster classifier so as to yield cluster weights for cluster ranking. Finally, informative clusters were selected based on the ranking of all clusters.

A cross-validation procedure was used to evaluate the performance of different mapping methods. That is, in each fold of the cross-validation, the original data were split into training and test data sets. The training data set was used for both selecting informative voxels (MIV) or clusters (MIC) and constructing a linear SVM classifier for classifying experimental conditions from the selected clusters. The test data set was then used to evaluate the predictive accuracy of the classifier constructed from the training data set.

### Partition of homogeneous clusters

Because voxels within a homogeneous cluster by definition show similar response profiles, we used an iterative algorithm of competitive region growing [21], [22] to partition the whole brain into homogeneous clusters. The similarity between two adjacent clusters (or voxels) was defined as:
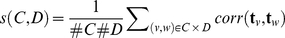
(1)where 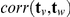 is the Pearson's linear correlation coefficient between the raw fMRI time series of voxel *v* and *w* (
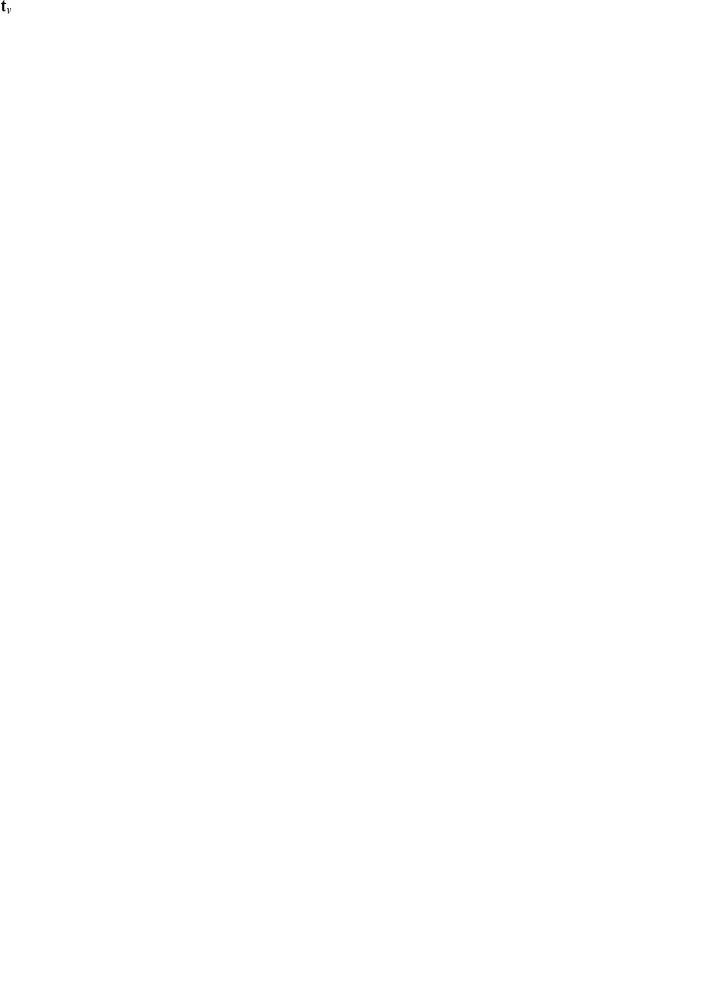
and 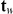) in cluster *C* and *D* respectively. #*C* and #*D* indicate the number of voxels in cluster *C* and *D* respectively. In particular, the algorithm constructed a set of candidate clusters starting from each voxel as a seed cluster. At each step, every pair of adjacent clusters that satisfied a mutual nearest neighbor principle was merged, and was then replaced by the merged cluster in the candidate set. That is, two adjacent clusters were merged if the correlation between their fMRI responses was the highest as compared to that between any pair formed with one of the two clusters and other neighbors. A merged cluster was removed from the set when its size reached a preset threshold (i.e., 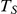). The algorithm stopped when there was no cluster in the set (i.e., all voxels were merged into homogeneous clusters whose size reached the threshold 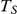) or no pair of clusters remaining in the candidate set satisfied the mutual nearest neighbor principle. Thus, the size of clusters ranged from 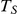 to 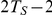. The partition procedure resulted in a set of 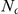 homogeneous clusters 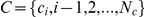.

### Univariate within-cluster summation

A straight-forward method to summarize information within a homogeneous cluster was to spatially average voxel responses (i.e., a univariate within-cluster summation for mapping informative clusters, or uMIC), similar to the regions of interest approach in a univariate analysis. The assumption was that the fMRI responses of voxels within a homogeneous cluster are sampled from an independent and identical distribution. Thus, a multi-voxel pattern 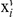 was transformed to a multi-cluster pattern 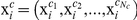 according to:
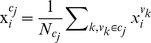
(2)where 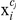 is the mean response of cluster 
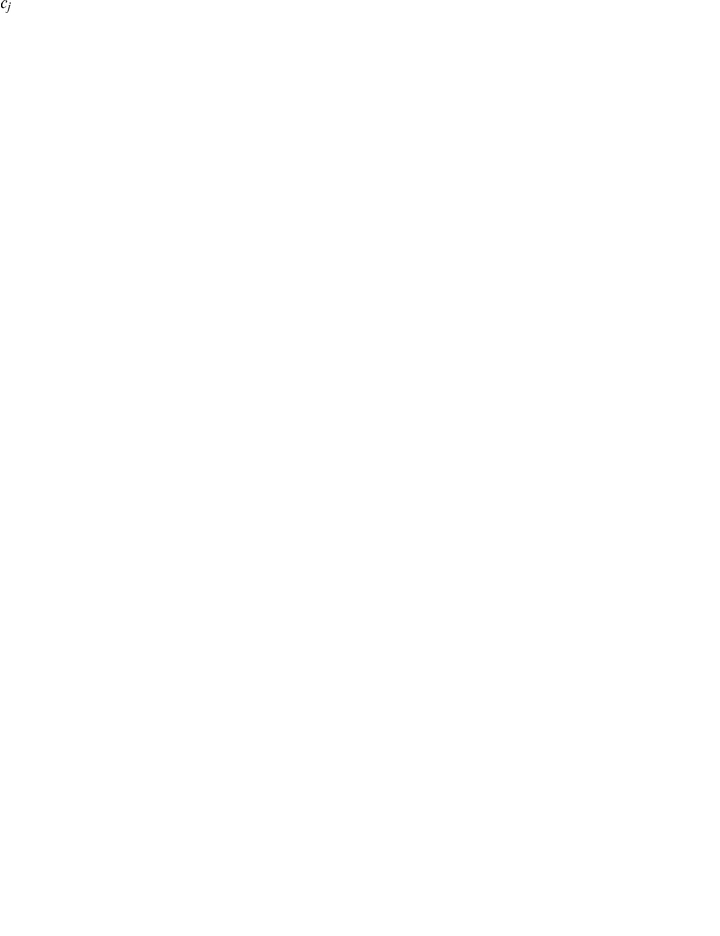
, 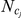 is the number of voxels in cluster 
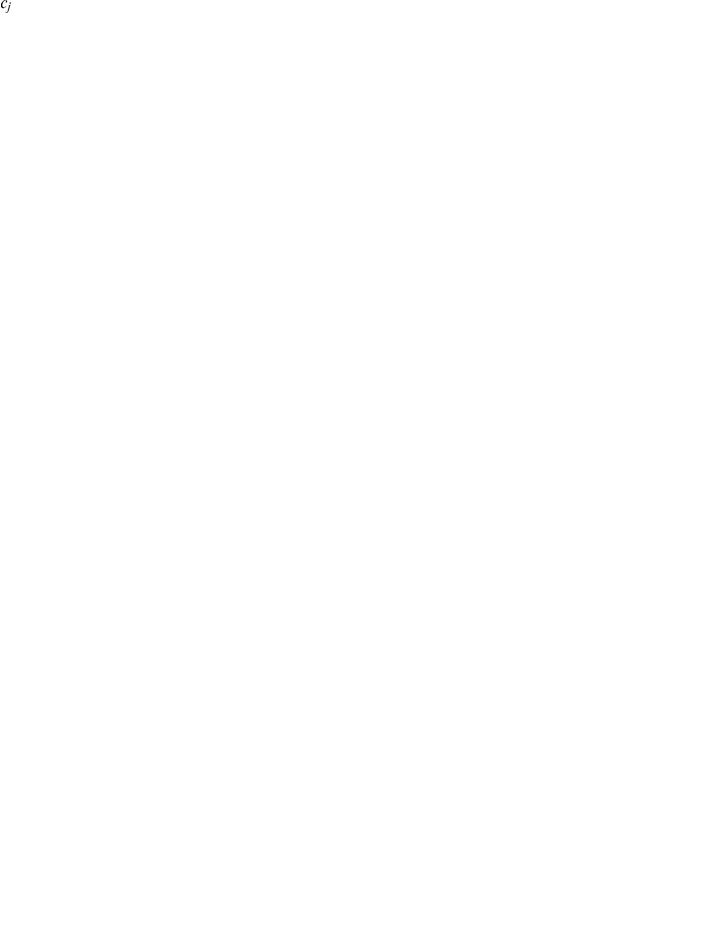
, and 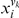 is the response of voxel 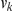 in pattern 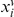.

### Multivariate within-cluster summation

In contrast to the univariate summation, multivariate methods can capture fine-scale information embedded in the multi-voxel pattern (i.e., a multivariate within-cluster summation for mapping informative clusters, or mMIC) by relaxing the independent and identical distribution assumption. Here we used Gaussian Naïve Bayesian (GNB) [3], [6] to summarize the multi-voxel pattern within a homogeneous cluster, which assumes that voxels within a homogeneous region are sampled from an independent but non-identical distribution. Specifically, we constructed a GNB classifier for each cluster respectively, which summed up the within-cluster multi-voxel patterns along with their corresponding condition labels from the training data. The output of the GNB classifiers (i.e., GNB discriminants) was taken as a within-cluster summation. For a multi-voxel pattern 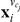 within cluster 
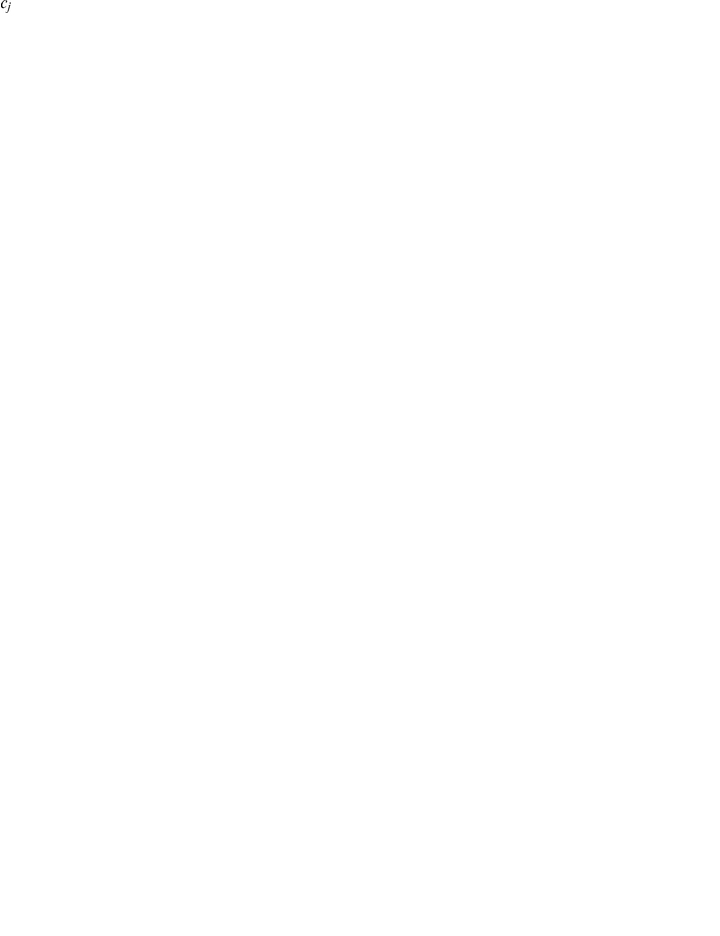
, the GNB discriminant is derived as:
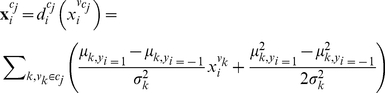
(3)where 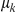 and 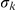 are computed for each voxel. The outputs (i.e., GNB discriminants) of all clusters constructs a multi-cluster pattern 
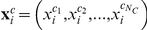
.

### Linear Support Vector Machine for ranking clusters/voxels

To identify informative clusters, discriminative weights were derived from linear Support Vector Machine (SVM) [23] that was trained on cluster patterns from either the uMIC or mMIC. Consider a pattern set 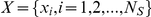 and its corresponding label set 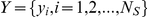, where 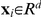 (*d* is the number of clusters) and 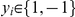 for binary cases, a linear SVM model learns a *d*-dimensional discriminant **w** to minimize
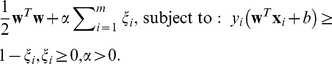
(4)


To classify a new pattern 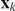, the label of 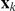 is denoted by 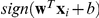. Therefore, the contribution of the *j*th cluster to the classification can be measured and ranked by the absolute value of its corresponding discriminative weight 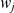. That is, if a cluster has a higher weight, it makes more contributions and thus carries more information to decode experimental conditions [8], [24]. Note that in the MIV, the whole brain multi-voxel pattern set 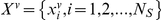 rather than the multi-cluster pattern set 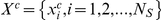 was used where the voxels with highest discriminative weights are then selected as informative ones. Other multivariate methods such as linear discriminant analysis (LDA) [18] or sparse logistic regression (SLR) [16] are also capable of serving as multivariate ranking methods as the SVM. We also constructed linear SVM classifiers on the mapped informative clusters to evaluate the predictive accuracy.

### Data descriptions and analysis

Simulated fMRI data

#### Data generation

The simulated fMRI data sets were created by following the procedure described in Kriegeskorte et al. [25]. The fMRI data (TR = 2s, matrix = 32×32, 5 slices, voxel size = 3×3×4 mm) were generated to simulate a blocked-design experiment consisting of two conditions. The experiment contained eight runs, each of which contained three 32-second blocks per condition separated by 16-second fixation blocks. Condition-related effects were set in two realistically shaped regions, with Gaussian white noise spatial patterns generated for each condition. The size of the informative regions varied at four levels: 15, 30, 60, and 120 voxels. There was no effect outside the preset informative regions. The time courses of BOLD signal were generated by convolving the experiment design with the canonical hemodynamic response function, overlapped with spatiotemporal Gaussian white noise that was spatially smoothed with a Gaussian kernel of 4 mm full width at half maximum. Four levels of functional contrast-to-noise ratios (CNR) (i.e., 0.05, 0.10, 0.15, and 0.20) were used in the simulation. The CNR was defined by spatially averaging maximum absolute activity level of signals within the informative regions divided by the temporal standard deviation of noise. The generation of simulated data was repeated 20 times for each combination of the parameters. That is, there were 20 (repetitions) ×4 (CNR levels) ×4 (region sizes) simulated fMRI data sets in total.

#### Data analysis

Partition of homogeneous regions and multivariate analyses were done with in-house code. To evaluate the influence of minimal cluster size (*T_S_*), performance metrics of the MIC were evaluated at different values of *T_S_* (i.e., 8, 15, 25, and 40).

The partition of homogeneous regions was carried out with raw time series, whereas the multi-voxel patterns used for multivariate mapping were created by shifting the time series by three volumes (i.e., 6 s) to account for the hemodynamic delay and then by averaging the signal intensity within blocks for each voxel. Thus there were 8 (runs) ×3 (blocks) samples for each condition. The extracted multi-voxel patterns with their corresponding experimental condition labels were fed into a four-fold cross-validation of both the MIC and MIV mappings and classification. This process was repeated 10 times to achieve stable results. In each fold, the mapping of informative regions and the training of classifier were carried out with 3/4 of the data, whereas the predictive accuracy of the trained classifier was tested with the rest 1/4 of the data.

For each of 4×4 combinations of the parameters, an ROC curve was produced by plotting sensitivity against 1-specificity in detecting the preset informative voxels at different thresholds of discriminative weights. Area under the ROC curve was used to evaluate the overall performance of these two mapping methods so as to examine to what extent both sensitivity and specificity were achieved [26], [27]. Moreover, clusters/voxels were ranked based on their discriminative weights, and the most discriminative ones were selected to construct ten feature levels, each of which corresponded to the different number of the most discriminative voxels ranging from 100 to 3844 voxels. The number of voxels was determined in a geometric progression fashion (i.e., 100, 150, 225, …, 3844). The predictive accuracy and robustness of mapping were assessed at these feature levels accordingly. Maximum predictive accuracy and the averaged robustness of mapping along the ten feature levels were submitted to statistical analyses.

In evaluating the robustness of functional mapping, we focused on within-subject variance because the selection of informative voxels or clusters in different folds varied significantly. Thus the robustness of mapping was defined as an averaged overlap rate among voxels selected in the four cross-validation folds. The overlap rate between voxels was quantified with a set-wise similarity metric [28]:
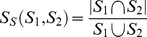
(5)where 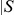 indicates the number of voxels in set *S*. 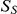 ranges from 0 when there is no overlap at all to 1 when the two sets of voxels were completely overlapped.

Real fMRI data

#### Data acquisition

The real fMRI data were collected from an object recognition experiment consisting of stimuli from six object categories: male faces, female faces, houses, scenes, cars, and chairs. Ten subjects (age 20–25; 2 males) participated in the study. All subjects were right-handed, and had normal or correct-to-normal vision. Written informed consent was obtained from each subject before participation. The study was approved by the IRB of Beijing Normal University.

Each subject participated in a single session consisting of twelve blocked-designed runs. Each run contained a total of six 32-second stimulus blocks, one stimulus category each, interleaved with seven 16-second fixation blocks, which gave 12 blocks (i.e., examples) for each category. Each stimulus block contained 40 trials, with a 300 ms stimulus display and a 500 ms blank display per trial. The stimuli were presented near fixation, and their positions were jittered across trials to prevent repetitive suppression of BOLD signals. Each of 20 stimulus exemplars from a category was presented twice in a block. The sequence of exemplars was pseudo-randomized, and four immediate repetitions were added to each block. The subjects were instructed to pay attention to each stimulus and to press a button when a repetition was detected (i.e., 1-back task).

Data were acquired on a Siemens 3T Trio scanner (MAGENTOM Trio, a Tim system) with a 12-channel phased-array head coil at BNU Imaging Center for Brain Research, Beijing. T2*-weighted gradient-echo, echo-planar images (EPI) of the whole brain were collected (TR = 2s, TE = 30 ms, FA = 90 degrees, FOV = 192×192 mm, matrix = 64×64, 25 slices, voxel size = 3×3×4 mm). Structural images were acquired with MPRAGE, an inversion prepared gradient echo sequence (TR/TE/TI = 2.53s/3.45ms/1.1s, FA = 7deg, voxel size = 1×1×1 mm). The structural images were used in registering the functional data to cortical surfaces and generating a mask of gray matter with Freesurfer [29], [30].

#### Data analysis

Preprocessing and univariate analyses of the fMRI data were conducted with the Freesurfer functional analysis stream (FS-FAST, http://surfer.nmr.mgh.harvard.edu). Preprocessing of the fMRI data involved motion correction and grand-mean intensity normalization.

After preprocessing, the univariate analysis was performed to localize regions selective for faces, houses and objects, which were later used in comparison with regions mapped by the multivariate analysis. In the univariate analysis, the fMRI data were spatially smoothed with a Gaussian kernel of 6 mm full width at half maximum. The BOLD responses were modeled by convolving a boxcar function with canonical hemodynamic response function. A general linear model was used to estimate the functional contrasts (i.e., face-selective regions: male and female faces versus the rest object categories; place-selective regions: houses and scenes versus the rest; object-selective regions: cars and chairs versus the rest).

For multivariate analyses, only the voxels within the anatomical mask of gray matter generated by Freesufer with the structural images were included. Linear detrending within runs was conducted for each voxel on the preprocessed fMRI data with no spatial smoothing. The remaining analyses were the same as those performed in the simulated data.

## Results

### Simulated fMRI data

**Figure 2:**
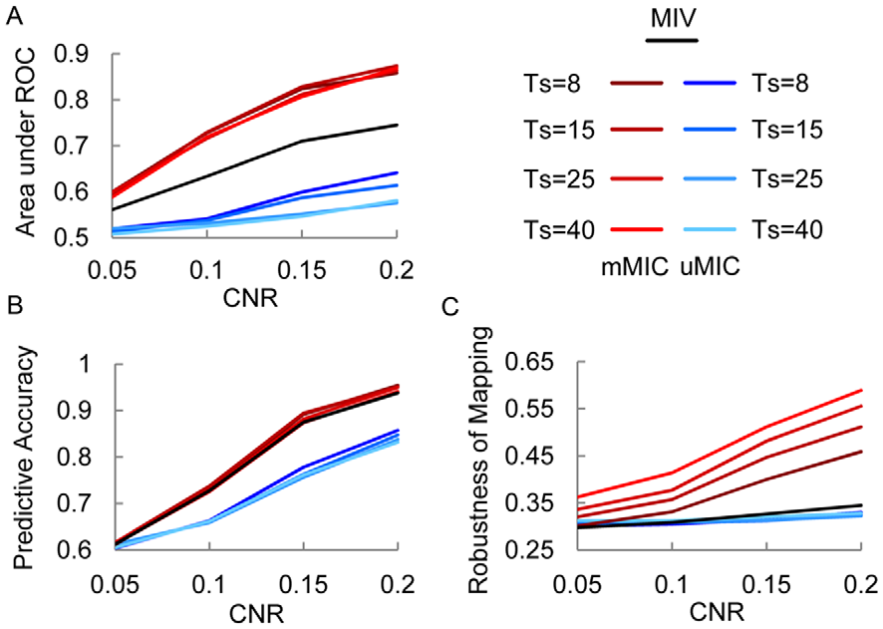
Performance of the MIC and the MIV in the simulated fMRI data. A) Area under ROC in the simulated fMRI data under various CNR averaged over 20 repetitions of data generation and different sizes of informative regions. B) Predictive accuracy and C) robustness of mapping.

We first evaluated the performance of the mapping methods by calculating area under ROC at various CNR. Because the size of the preset informative regions did not affect the results across different methods (Figure S1), the results at different sizes were pooled together by averaging to simplify statistical analyses. Under all levels of the CNR and 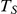, the area under ROC in the mMIC was significantly higher than that in the MIV (all *p*s<0.05), and the area under ROC in the MIV was significantly higher than that in the uMIC (all *p*s<0.05) (Figure 2A). The predictive accuracy showed a similar pattern (Figure 2B), with the predictive accuracy in the mMIC was the highest, and that in the uMIC was the lowest, except that at the CNR of 0.05 the predictive accuracies of all methods were near the chance level (i.e., 0.50) (Table S1). More importantly, as for robustness of mapping the mMIC outperformed both the MIV and the uMIC (all *p*s<0.01) at all levels of the CNR and 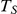 (Figure 2C), whereas the robustness of the uMIC in mapping did not significantly differ from that in the MIV (Table S2).

Thus, the mMIC outperformed the MIV in robustness of localizing the informative voxels with slightly better or at least identical predictive accuracy, whereas uMIC showed the worst performance in both predictive accuracy and the robustness of mapping. In the next step, we used real fMRI data to further test the feasibility and validity of the mMIC. Because the performance of the uMIC was lower in both classification and mapping, we did not include it in the further analysis. In addition, because the mMIC was relatively tolerant of the change of the preset cluster size (i.e., Ts), only one Ts was chosen in analyzing the real fMRI data. The 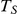 was set to 15 voxels which showed the best trade-off between predictive accuracy and robustness of mapping suggested by the results from the simulated data (Figure 2 & Figure S1).

### Real fMRI data

Information on the partition of homogeneous clusters in the real fMRI data was summarized in Table 1. Around 25,000 voxels were grouped into 1,200∼1,500 homogeneous clusters; less than 5% of voxels was not grouped into any clusters and thus were excluded from further analysis. The distribution of averaged within-cluster temporal correlation coefficients between voxels was summarized in a histogram across all subjects with a mean coefficient at 0.18 (Figure S2). All partitioned homogeneous clusters of the representative subject were shown in a slice view, with randomized pigmentation for different clusters (Figure S3).

**Table 1:** Information on partition of homogenous clusters.

Subjects	BL	HP	JY	LY	LJ	TM	XM	XQ	XR	ZY
nHC	1376	1479	1229	1370	1364	1405	1368	1288	1491	1315
nVox	25451	27350	22479	25221	25407	25820	25300	23946	27426	24590
nVoxEx	1063	1115	926	1046	1024	1161	1049	1034	1259	1117
nVoxEx/nVox	4.18%	4.08%	4.12%	4.15%	4.03%	4.5%	4.15%	4.32%	4.59%	4.54%

nHC – number of homogeneous clusters; nVox – number of voxels in the structural mask of gray matter; nVoxEx – number of voxels not partitioned to any clusters.

**Figure 3:**
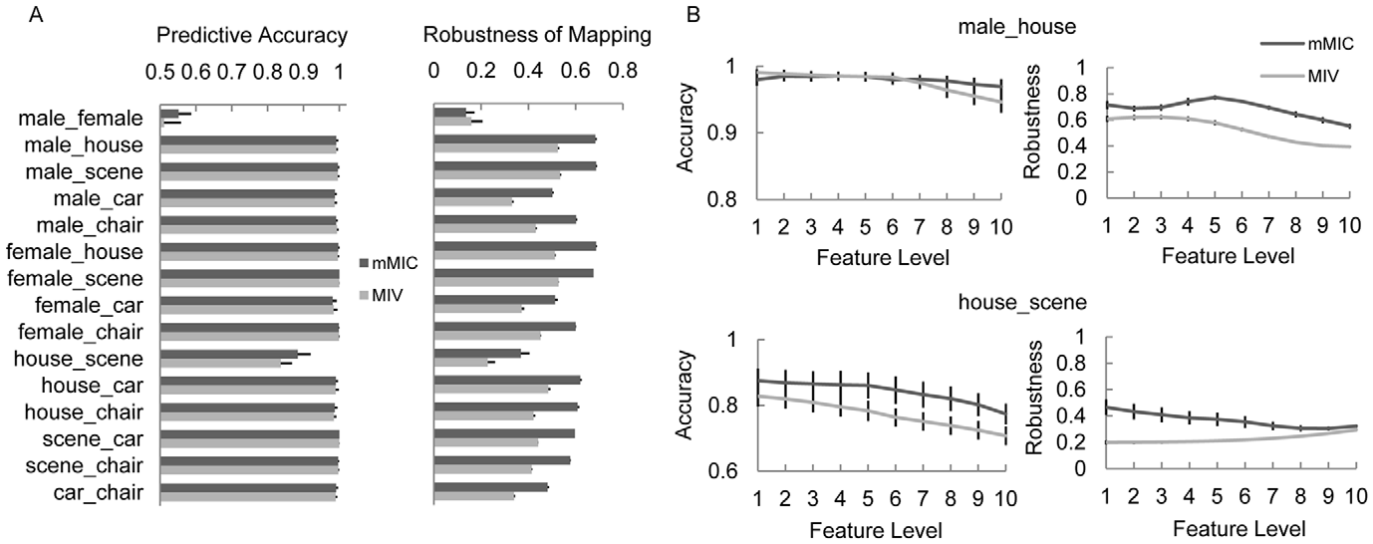
Performance of the mMIC and the MIV in the real fMRI data. A) Predictive accuracy (left) and robustness of mapping (right) for all 15 contrasts. Dark bar: mMIC; light bar: MIV. B) Predictive accuracy and robustness of mapping at 10 feature levels in two representative contrasts (male_house: male faces versus houses; house_scene: houses versus scenes). Error bars indicate standard error of mean across subjects.

Because there were six object categories, the classification between any two categories gave rise to fifteen contrasts in total. Predictive accuracy and the robustness of mapping were calculated for each contrast respectively. The pattern of predictive accuracy was similar to that in simulated data: the mMIC achieved higher or comparable predictive accuracy compared to the MIV (Figure 3A, left panel). The reason that the difference in predictive accuracy between the mMIC and the MIV did not reach significance likely resulted from the fact that there was abundant discriminative information in category-dependent patterns. In addition, the classification accuracy in differentiating male versus female faces was higher than the chance level, consistent with a recent study where MVPA analyses were used to differentiate other finer sub-category distinctions, such as faces of different age [31]. High resolution fMRI may help discriminate these fine differences [32].

More importantly, the mMIC outperformed MIV in all the contrasts in mapping (Figure 3A, right panel, all *p*s<0.05). Similar results were found for each of the 15 contrasts at each of the 10 feature levels. Figure 3B illustrates two representative contrasts: male faces vs. houses with near perfect predictive accuracy, and houses vs. scenes with relatively lower predictive accuracy, again showing that the mMIC generated higher (or comparable) predictive accuracy and robustness of mapping than the MIV at all feature levels. Note that the application of homogeneous information embedded in fMRI responses among voxels in identifying clusters is necessary, because when this information was ignored the performance was significantly deteriorated (Figure S4). It has been recently shown that spatial smoothing greatly improves the performance of the MIV [33], [34], [35], [36], which is replicated in this study (Figure S5). However, the mMIC was superior to the MIV with spatial smoothing, suggesting that the hierarchical framework also contributes significantly.

**Figure 4:**
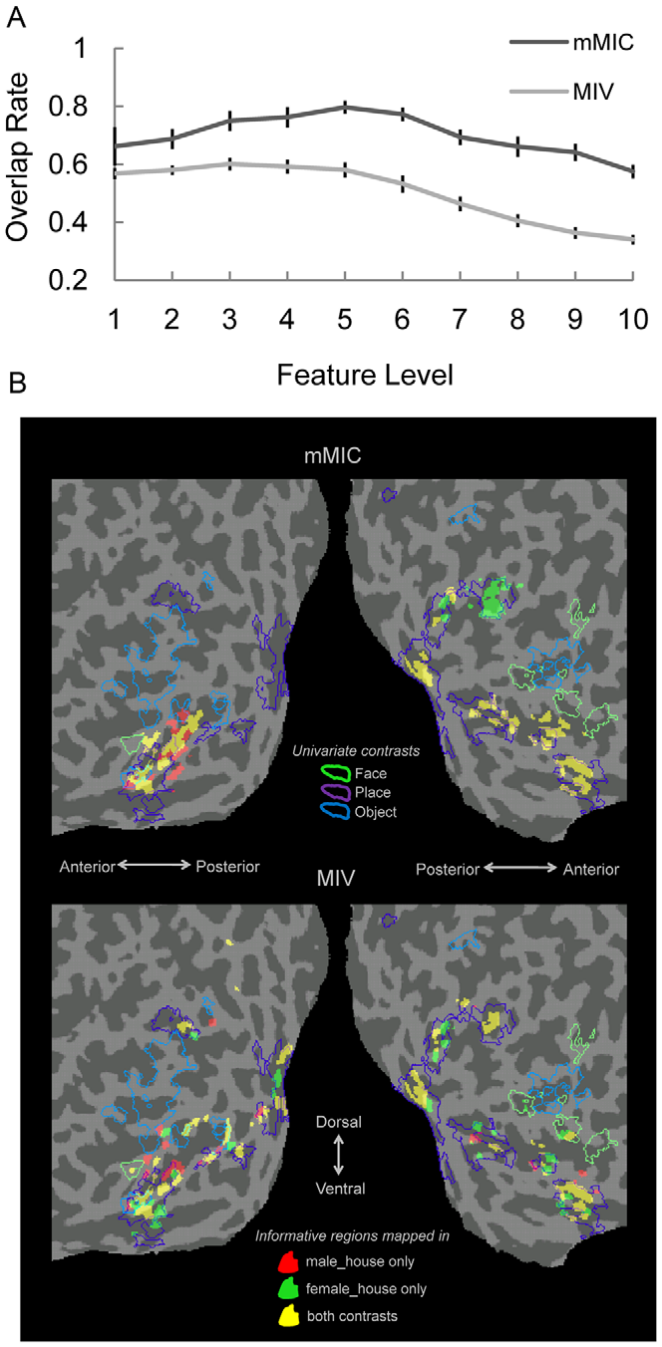
Overlap between informative regions mapped for female and male faces. A) Overlap rate between informative regions mapped in the contrasts of male faces vs. houses and those in the contrast of female faces vs. houses. Error bars indicate standard error of mean across subjects. B) Distribution of the overlap between informative regions for female and male faces by the mMIC and the MIV respectively. The size of regions was chosen to match the feature level at which the best prediction performance was achieved, which was set to the same for both methods and in both contrasts. For comparison, face-, place- and object-selective regions localized by the univariate analysis (p<10^−6^, uncorrected) were shown in colored contours as a reference.

We next examined whether the regions mapped by the mMIC were related to brain functions. To examine the validity of the functional mapping, we utilized the fact that informative voxels involved in processing male faces shall be largely overlapped with those in processing female faces simply because they belong to the same category. The overlap rate between informative regions mapped in the contrasts of male faces (versus houses) and female faces (versus houses) for the mMIC and the MIV was calculated respectively. The overlap rate was significantly larger in the mMIC than that in the MIV at almost all feature levels (*p*s<0.05 at all feature levels except the first one with the smallest number of voxels) (Figure 4A). The overlap of regions mapped in these two contrasts was shown for the mMIC and the MIV respectively (Figure 4B), which clearly showed that the informative regions encoding female and male faces were highly localized and were largely overlapped in the mMIC, whereas those mapped by the MIV were distributed and shared much fewer overlapped voxels.

## Discussion

MVPA has been successful in discriminating mental states by investigating subtle information embedded in multi-voxel pattern of fMRI activities. Here we proposed a new method to improve the validity and reliability of MVPA in localizing the discriminative patterns by taking the local homogenous clusters as the basic unit in the mapping (i.e., mapping informative clusters, MIC). The results from both the simulated data and the fMRI data showed that the MIC via multivariate within-cluster summation (mMIC) exceeded the voxel-based MVPA methods (MIV) in functional brain mapping.

The advantage of the mMIC over the MIV is non-trivial. First, mapping informative regions at the level of local homogeneous clusters not only agreed with the spatiotemporal nature of fMRI data, but also echoed the findings from neurophysiological and neuroimaging studies. That is, neurons with similar response profiles are usually clustered together, forming relatively homogeneous cortical regions [37], [38], [39], [40]. Second, the mMIC significantly reduced the dimension of fMRI data by taking homogeneous clusters, rather than voxels, as the basic unit in the MVPA analysis. Finally, the hierarchical framework ensured that both local multi-voxel patterns within a cluster and global multi-cluster patterns within the brain were used so that information at different spatial scales was taken into account in the MVPA analysis.

Our method is in line with previous studies on mapping information regions with MVPA. To address the spatiotemporal correlation of fMRI data, Carroll et al. [17] used the Elastic Net [41] to assign similar weights to correlated features, and van Gerven et al. [42] introduced groupwise regularization to select clusters. In addition, the hierarchical framework has been adopted in studies where the brain was first segmented into regions based on an anatomical atlas for constructing local classifiers, which were later combined [43], . Our approach extends these prior works in three aspects. First, the spatiotemporal correlations in BOLD fluctuations were taking into account in an explicit manner. Second, the clusters were derived based on the task-specific fMRI data rather than anatomical atlas. Finally, our method searched for informative clusters all over the brain, rather than restricted to a few regions.

Although our method is better than voxel-based MVPA methods, several issues remain. First, although the mMIC was better in discriminating some experimental conditions (e.g. houses versus scenes) than the MIV, the predictive accuracy of the mMIC in general was not significantly better, possibly because the accuracy in other experimental contrasts was at ceiling. Future experiments are needed to systematically compare these two methods in predictive accuracy when it is not at ceiling (i.e., experimental conditions are difficult to be differentiated, like houses versus scenes). Second, the preset size of homogeneous clusters (i.e., 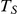) was thought to affect the performance of the mMIC, depending on how well the preset size matched the actual size of functional regions of interest. However, results from the simulated data showed that the mMIC was relatively tolerant of the change of the preset size of homogeneous clusters. This finding suggests that the mMIC might be suitable for a wide range of functional mapping tasks, irrespectively to the size of functional regions. On the other hand, the fixed size of homogeneous clusters may lower the performance of the mMIC; therefore, future algorithms with varying cluster size that is adaptive to the actual size of functional regions may help improve the performance. In this study, we preset the size of clusters for the purpose of unbiased comparisons among clusters. Instead, we could use the degree of local homogeneity in defining clusters so that the size of clusters can be adaptively adjusted based on the functional characteristics of different cortical regions. Third, we used the GNB classifier to extract the fine multi-voxel pattern in a local cluster based on the assumption that the fMRI responses of neighboring voxels were independent. Classifiers, such as regularized LDA, do not rest on this assumption; therefore, they might be more accurate in extracting information embedded in local multi-voxel patterns.

## Supporting Information

Figure S1 **Performance of the MIC and the MIV in the simulated fMRI data at different size of preset homogeneous regions.** The results from different size of preset homogeneous regions were shown in four columns respectively. The pattern was similar across region size. For area under ROC, the performance of the mMIC were significantly better than the MIV (*p*s<0.05 in all conditions except for the lowest CNR (0.05) with two smallest region sizes (15 and 30)), and the performance of the MIV was better than that of the uMIC (*p*s<0.05 in all conditions except for the lowest CNR with two smallest region sizes). For the predictive accuracy, the mMIC was comparable to or slightly better than the MIV, and the MIV was significantly better than the uMIC (*p*s<0.05 under all conditions except for the lowest CNR). For robustness of mapping, there was no significant difference between the MIV and uMIC, and the mMIC was significantly better than either the MIV or uMIC (all *ps*<0.05).
(TIF)

Figure S2 **Within-cluster temporal correlations in real fMRI data.** To examine the within-cluster homogeneity of clusters partitioned by the region growing algorithm, we calculated the mean vale of inter-voxel temporal correlation coefficients between fMRI time courses of all pairs of voxels within the same cluster. The histogram shows the distribution of the within-cluster correlations from all clusters and from all ten subjects.
(TIF)

Figure S3 **Slice view of homogeneous clusters.** Homogeneous clusters of a representative subject are shown in a slice view. The homogeneous clusters were partitioned by the iterative region growing method from all gray matter voxels of this subject. Colors were used to mark different homogeneous clusters.
(TIF)

Figure S4 **Performance of the mMIC on homogeneous clusters versus non-homogeneous clusters.** To examine whether the homogeneous clusters help improve the performance of the mMIC, we partitioned the brain into non-overlapped cubic-shaped clusters, irrespectively to the embedded correlations among the BOLD signals of voxels. The rest procedure, including the sum-up of the within-cluster patterns and the classification of multivariate GNB discriminants, was the same as the mMIC. The modified method is referred as the mMICc for simplicity. Three levels of cluster size in the mMICc were chosen (i.e., 18, 48, and 75 voxels per cluster, labeled as Size I, II, and III) to match the mean size of the homogeneous clusters with Ts of 15, 40, and 60 in the mMIC. To simplify the comparison, the performance on each experimental contrast was pooled together, and the averaged performance was then submitted to a two-way ANOVA with factors as homogeneity (homogeneous clusters versus cubic-shaped clusters) and cluster size. We found that 1) for the predictive accuracy, the performance was similar at all cluster size tested, with a slight gain in using homogeneous clusters (Left); 2) for the robustness of functional mapping, the mMIC based on homogeneous clusters was significantly better in overall (F(1,9) = 56.7, *p*<0.001), especially at larger cluster sizes (cluster size II, *t*(9) = 4.5, *p* = 0.001; cluster size III, *t*(9) = 2.3, *p* = 0.04) (Right). In addition, because neighboring voxels usually share similar response characteristics, the cubic-shaped clusters likely contained large amount of homogeneous voxels. Thus, it is not surprising that its performance was significant better than that of the MIV (gray dotted line) that completely ignored correlations in BOLD signals among voxels at all sizes (predictive accuracy: all *p*s<0.05; robustness of mapping: all *p*s<0.001). Taken together, our result suggests that the homogeneous information embedded in BOLD signals among voxels helps improve the robustness of functional mapping without compromising the predictive accuracy. Error bars indicate standard error of mean across subjects.
(TIF)

Figure S5 **The effect of spatial smoothing on MVPA analyses.** Recently, it has been shown that spatial smoothing is beneficial for MVPA analyses [33], [34], [35], [36]. Here we examined whether the spatial smoothing was a major factor driving the performance of the mMIC. To do this, we applied the MIV to spatially smoothed data with a Gaussian kernel of 6 mm full width at half maximum (henceforth called MIVs), and then compared the performance of the MIVs to that of the MIV and the mMIC based on unsmoothed data. A) Predictive accuracy and robustness of mapping. For experimental contrasts where the mMIC, MIV, and MIVs achieved near-perfect performance in predictive accuracy, there was no significant difference between them (all *t*s<1) (Left). For the contrast of male versus female faces, the MIVs was better than the MIV in predictive accuracy (*t*(9) = 2.06, *p* = 0.07), whereas there was no difference between mMIC and MIVs (*t*<1). For the contrast of houses versus scenes, the MIVs was inferior to the mMIC (t(9) = 2.11, *p* = 0.06), and there was no difference between the MIVs and the MIV (*t*<1). The robustness of functional mapping of the MIVs showed a similar pattern (Right). The MIVs outperformed MIV in all the contrasts (all *p*s<0.05). More critically, the MIVs was inferior to the mMIC in all the contrasts (all *p*s<0.05). B) Overlap between informative clusters mapped for female and male faces (versus houses). The functional validity of the MIVs, MIV, and MIC was examined by calculating the overlap rate between informative regions mapped with male faces (versus houses) and female faces (versus houses). Similarly, the overlap rate in the MIVs was significantly higher than that in the MIV (*p*s<0.05 at all feature levels except for the first four), and was significantly smaller than that in the mMIC (all *p*s<0.05 at all feature levels except the first and the seventh levels). Error bars indicate standard error of mean across subjects.
(TIF)

Table S1 **Predictive accuracy in the simulated data by uMIC, mMIC, and MIV, under various settings of CNR and Ts.**
(DOC)

Table S2 **Robustness of mapping in the simulated data by uMIC, mMIC, and MIV, under various settings of CNR and Ts.**
(DOC)
