# Correction: Mapping Informative Clusters in a Hierarchial Framework of fMRI Multivariate Analysis

**DOI:** 10.1371/annotation/0cca502f-3308-44a1-9536-be92153df9ea

**Published:** 2011-02-01

**Authors:** Rui Xu, Zonglei Zhen, Jia Liu

The word "Hierarchical" is misspelled in the article title. The correct title is: Mapping Informative Clusters in a Hierarchical Framework of fMRI Multivariate Analysis. The correct citation is: Xu R, Zhen Z, Liu J (2010) Mapping Informative Clusters in a Hierarchical Framework of fMRI Multivariate Analysis. PLoS ONE 5(11): e15065. doi:10.1371/journal.pone.0015065

