# Supplementary figures and images for: Mapping Informative Clusters in a Hierarchial Framework of fMRI Multivariate Analysis
Source: PLoS One. 2010 Nov 30;5(11):e15065. doi: 10.1371/journal.pone.0015065 (PMC2994831; doi:10.1371/journal.pone.0015065)

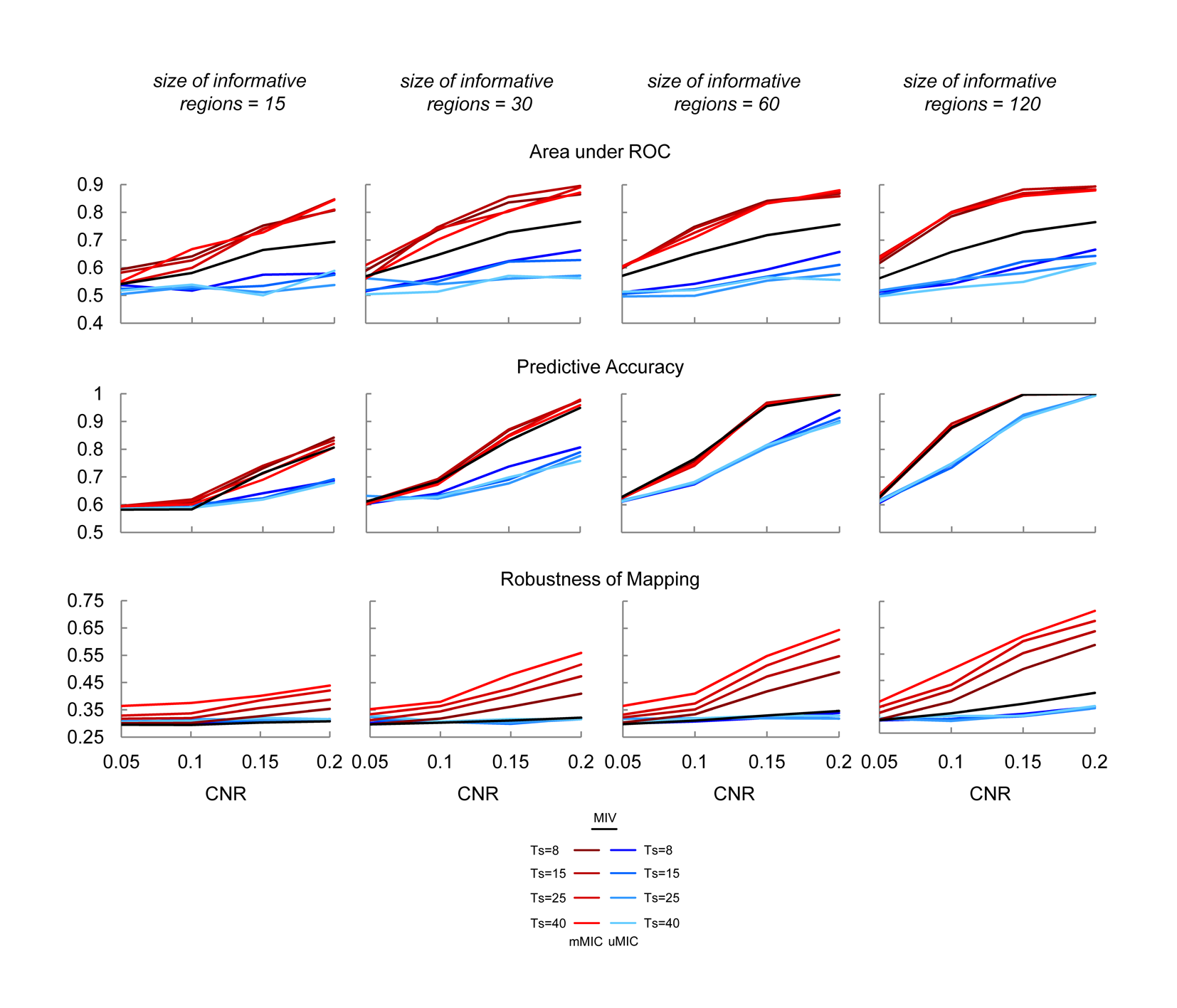

Supplement: Figure S1 — Performance of the MIC and the MIV in the simulated fMRI data at different size of preset homogeneous regions. The results from different size of preset homogeneous regions were shown in four columns respectively. The pattern was similar across region size. For area under ROC, the performance of the mMIC were significantly better than the MIV (ps<0.05 in all conditions except for the lowest CNR (0.05) with two smallest region sizes (15 and 30)), and the performance of the MIV was better than that of the uMIC (ps<0.05 in all conditions except for the lowest CNR with two smallest region sizes). For the predictive accuracy, the mMIC was comparable to or slightly better than the MIV, and the MIV was significantly better than the uMIC (ps<0.05 under all conditions except for the lowest CNR). For robustness of mapping, there was no significant difference between the MIV and uMIC, and the mMIC was significantly better than either the MIV or uMIC (all ps<0.05). (TIF) [file pone.0015065.s001.tif]

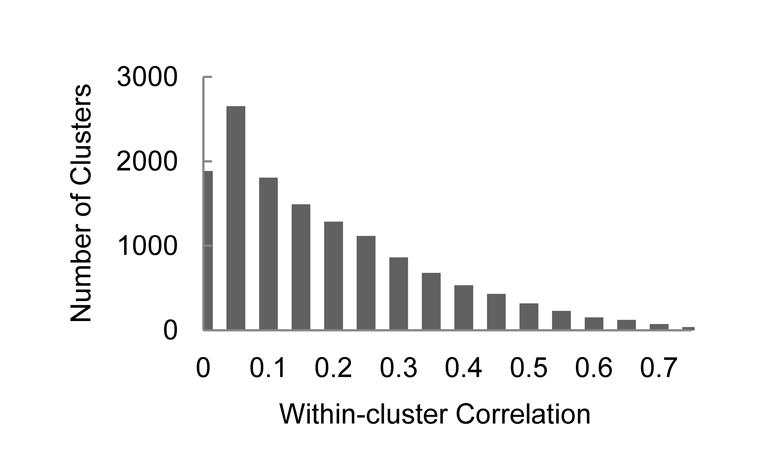

Supplement: Figure S2 — Within-cluster temporal correlations in real fMRI data. To examine the within-cluster homogeneity of clusters partitioned by the region growing algorithm, we calculated the mean vale of inter-voxel temporal correlation coefficients between fMRI time courses of all pairs of voxels within the same cluster. The histogram shows the distribution of the within-cluster correlations from all clusters and from all ten subjects. (TIF) [file pone.0015065.s002.tif]

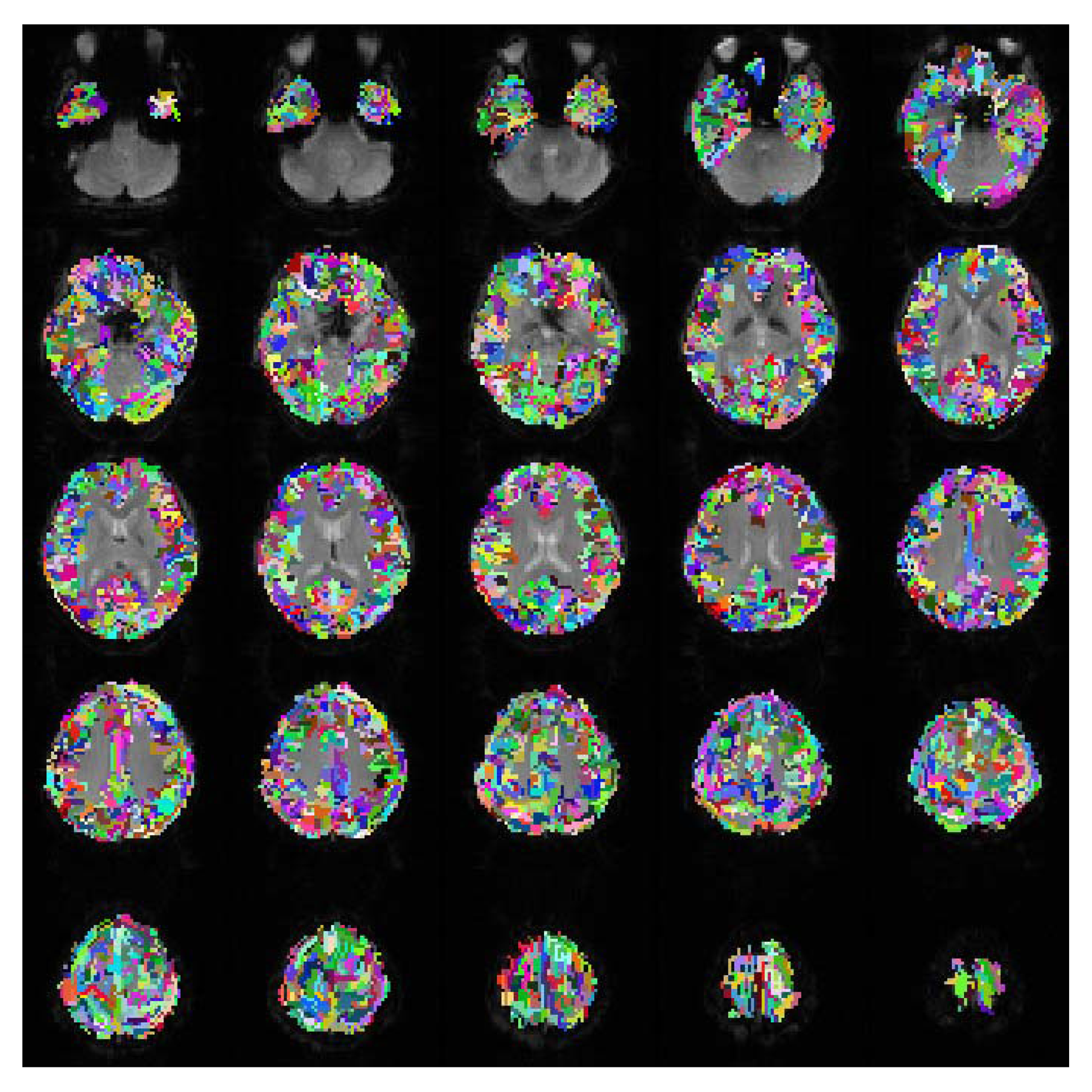

Supplement: Figure S3 — Slice view of homogeneous clusters. Homogeneous clusters of a representative subject are shown in a slice view. The homogeneous clusters were partitioned by the iterative region growing method from all gray matter voxels of this subject. Colors were used to mark different homogeneous clusters. (TIF) [file pone.0015065.s003.tif]

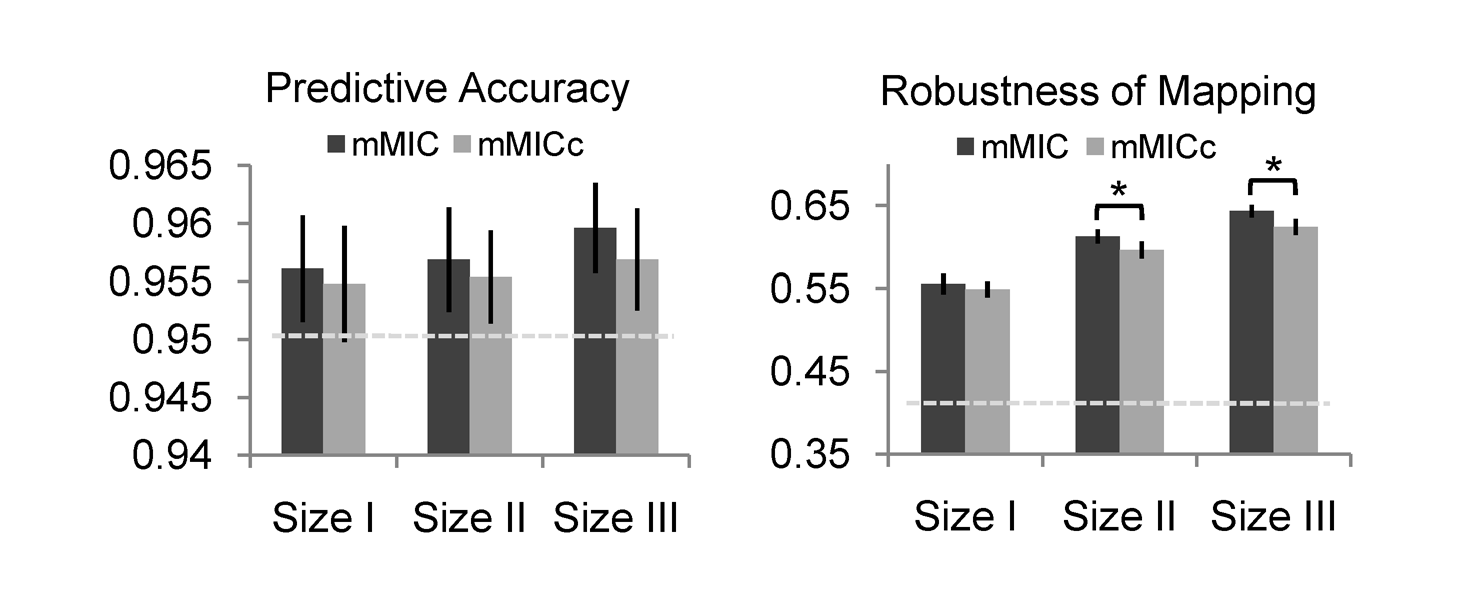

Supplement: Figure S4 — Performance of the mMIC on homogeneous clusters versus non-homogeneous clusters. To examine whether the homogeneous clusters help improve the performance of the mMIC, we partitioned the brain into non-overlapped cubic-shaped clusters, irrespectively to the embedded correlations among the BOLD signals of voxels. The rest procedure, including the sum-up of the within-cluster patterns and the classification of multivariate GNB discriminants, was the same as the mMIC. The modified method is referred as the mMICc for simplicity. Three levels of cluster size in the mMICc were chosen (i.e., 18, 48, and 75 voxels per cluster, labeled as Size I, II, and III) to match the mean size of the homogeneous clusters with Ts of 15, 40, and 60 in the mMIC. To simplify the comparison, the performance on each experimental contrast was pooled together, and the averaged performance was then submitted to a two-way ANOVA with factors as homogeneity (homogeneous clusters versus cubic-shaped clusters) and cluster size. We found that 1) for the predictive accuracy, the performance was similar at all cluster size tested, with a slight gain in using homogeneous clusters (Left); 2) for the robustness of functional mapping, the mMIC based on homogeneous clusters was significantly better in overall (F(1,9) = 56.7, p<0.001), especially at larger cluster sizes (cluster size II, t(9) = 4.5, p = 0.001; cluster size III, t(9) = 2.3, p = 0.04) (Right). In addition, because neighboring voxels usually share similar response characteristics, the cubic-shaped clusters likely contained large amount of homogeneous voxels. Thus, it is not surprising that its performance was significant better than that of the MIV (gray dotted line) that completely ignored correlations in BOLD signals among voxels at all sizes (predictive accuracy: all ps<0.05; robustness of mapping: all ps<0.001). Taken together, our result suggests that the homogeneous information embedded in BOLD signals among voxels helps improve the robustn [file pone.0015065.s004.tif]

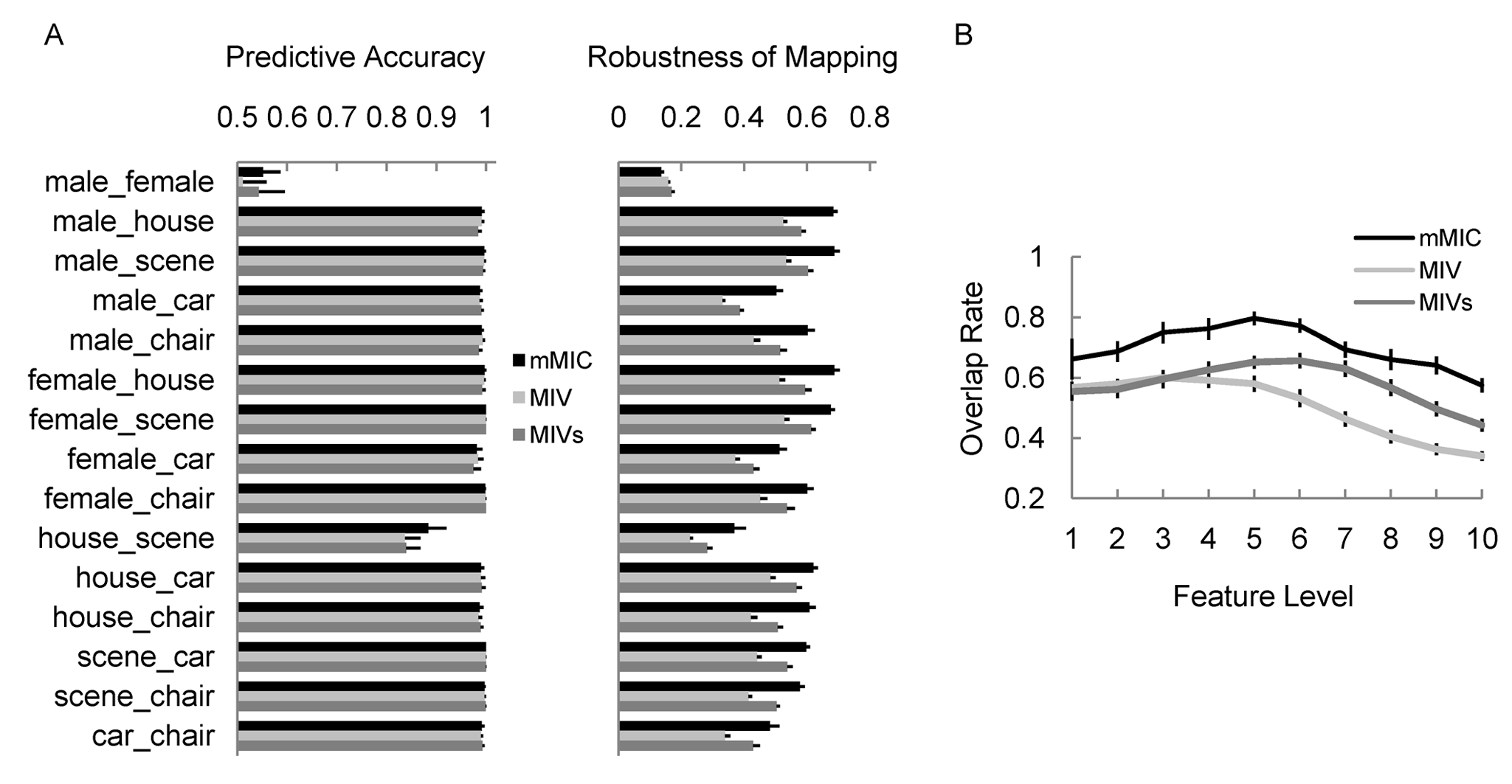

Supplement: Figure S5 — The effect of spatial smoothing on MVPA analyses. Recently, it has been shown that spatial smoothing is beneficial for MVPA analyses [33], [34], [35], [36]. Here we examined whether the spatial smoothing was a major factor driving the performance of the mMIC. To do this, we applied the MIV to spatially smoothed data with a Gaussian kernel of 6 mm full width at half maximum (henceforth called MIVs), and then compared the performance of the MIVs to that of the MIV and the mMIC based on unsmoothed data. A) Predictive accuracy and robustness of mapping. For experimental contrasts where the mMIC, MIV, and MIVs achieved near-perfect performance in predictive accuracy, there was no significant difference between them (all ts<1) (Left). For the contrast of male versus female faces, the MIVs was better than the MIV in predictive accuracy (t(9) = 2.06, p = 0.07), whereas there was no difference between mMIC and MIVs (t<1). For the contrast of houses versus scenes, the MIVs was inferior to the mMIC (t(9) = 2.11, p = 0.06), and there was no difference between the MIVs and the MIV (t<1). The robustness of functional mapping of the MIVs showed a similar pattern (Right). The MIVs outperformed MIV in all the contrasts (all ps<0.05). More critically, the MIVs was inferior to the mMIC in all the contrasts (all ps<0.05). B) Overlap between informative clusters mapped for female and male faces (versus houses). The functional validity of the MIVs, MIV, and MIC was examined by calculating the overlap rate between informative regions mapped with male faces (versus houses) and female faces (versus houses). Similarly, the overlap rate in the MIVs was significantly higher than that in the MIV (ps<0.05 at all feature levels except for the first four), and was significantly smaller than that in the mMIC (all ps<0.05 at all feature levels except the first and the seventh levels). Error bars indicate standard error of mean across subjects. (TIF) [file pone.0015065.s005.tif]
